# Examination of the importance of anger/irritability and limited prosocial emotion/callous-unemotional traits to understand externalizing symptoms and adjustment problems in adolescence: A 10-year longitudinal study

**DOI:** 10.3389/fpsyt.2022.939603

**Published:** 2022-09-29

**Authors:** Sébastien Urben, Stéphanie Habersaat, Julie Palix, Jörg M. Fegert, Klaus Schmeck, David Bürgin, Süheyla Seker, Cyril Boonmann, Marc Schmid

**Affiliations:** ^1^Division of Child and Adolescent Psychiatry, University Hospital of Lausanne (CHUV) and University of Lausanne, Lausanne, Switzerland; ^2^Department of Child and Adolescent Psychiatric Research, Psychiatric University Hospitals Basel (UPK), Basel, Switzerland; ^3^Institute of Forensic Psychiatry, University Hospital of Lausanne (CHUV), Lausanne, Switzerland; ^4^Department of Child and Adolescent Psychiatry and Psychotherapy, University Hospital of Ulm, Ulm, Germany; ^5^Department of Forensic Child and Adolescent Psychiatry, Psychiatric University Hospitals Basel (UPK), Basel, Switzerland

**Keywords:** emotions, self-regulation (SC 23180), adjustment problems, adolescents, young adults, longitudinal design

## Abstract

**Objective:**

Within a longitudinal study (10-year follow-up), we aim to examine the role of anger/irritability and limited prosocial emotion/callous-unemotional traits in predicting externalizing symptoms and adjustment problems in individuals formerly in youth residential care institutions.

**Method:**

These dimensions were assessed in 203 young adults, with baseline assessments during youth residential care and a follow-up 10 years later.

**Results:**

In general, emotional problems and psychopathological symptoms did not reduce over time. Analyses of regression revealed that a younger age at baseline, anger/irritability both at baseline assessment, and regarding their aggravation over time refer to significant predictors of the level of externalizing symptoms at 10-year follow-up (*R*^2^ = 0.431) and the worsening of externalizing symptoms over time (*R*^2^ = 0.638). Anger/irritability has been observed to be a significant predictors of both the level of adjustment problems at 10-year follow-up (*R*^2^ = 0.471) and its worsening over time (*R*^2^ = 0.656).

**Discussion:**

Our results suggest that dysregulation of anger/irritability is a key factor in the prediction of long-term externalizing symptoms and adjustment problems as well as its worsening over time. Possible implications for intervention and prevention are discussed.

## Introduction

### Externalizing symptoms and adjustment problems of youths in residential care

Recent studies showed that individuals who grew up in residential youth care were more at risk for a large array of psychological problems, including internalizing and externalizing symptoms (1–5). Indeed, growing up in residential youth care may lead to experience stress (6). Youths have to adjust to a new environment (i.e., residential youth care) as well as to cope with the reasons leading to the placement, such as instable, violent, neglecting, or inappropriate family environment (e.g., death of a parent, mental illness of a parent, abuse and neglect, and recurrent family conflicts). All of these factors, especially when youths do not have access to sufficient internal and external resources to cope with these situations, generate chronic stress [see, e.g., (7)]. Chronic stress was, in turn, related to the development of maladaptive coping and regulation strategies when confronted to a new situation of stress, such as anger outburst, violent behaviors, or excessive anxiety, which are known factors facilitating the development of persisting adjustment problems (5, 8–10).

Adjustment problems are defined in the literature as a large category of different features of dysfunction, including internalized symptoms such as anxiety, depression, withdrawal, and/or externalized symptoms such as aggressive, violent, or antisocial behaviors, significantly impacting the psychosocial wellbeing of an individual and their integration in the society (11–14).

In some individuals, externalizing, or more broadly adjustment, problems reach a peak at adolescence and gradually decline into adulthood following a natural desistance pathway, while in others, such difficulties may be more profoundly anchored in functioning, increasing the risk of developing psychiatric disorders or of settling in a criminal career (15–22). Factors predictive of long-term adjustment or externalizing problems are therefore an important area of research to foster preventive care strategies.

### Emotional features of youths with externalizing or adjustment problems

One important notion that might help to understand why an individual follows a specific trajectory is the presence of specific emotional features (18–22). Indeed, difficulties in regulating emotions such as anger/irritability or, on the opposite, difficulties in accessing or feeling emotions (limited prosocial emotions/callous-unemotional traits—LPE/CU) were evidenced in individuals exposed to chronic life stress environment and were shown to be associated with externalizing symptoms (23–26). In particular, high levels of anger/irritability in children were observed to be associated with aggression and conduct problems (27–29) and emotional lability later in life (30). The fifth edition of *the* Diagnostic and Statistical Manual of Mental Disorders (DSM-V) included an “*anger/irritability*” component in the diagnostics of oppositional defiant disorder and conduct disorder [e.g., (31)]. In addition, chronic anger was observed to be a precursor of antisocial personality disorder (32). LPE/CU traits were related to psychopathic personality traits (33–36) and were shown to be predictive of externalizing behaviors in adulthood (37, 38).

### Cumulative effects

Although there is evidence of a co-occurrence of anger/irritability and LPE/CU in different DSM-V diagnostic categories related to externalizing behaviors (39, 40), only few studies examined both characteristics conjointly. However, the cumulative effects of negative factors might increase the risk of impaired life trajectories (41). In a previous cross-sectional study of our team (25), we examined the role of age of onset of externalizing behaviors, anger/irritability, and LPE/CU in antisocial behaviors (delinquency and externalizing symptoms) in institutionalized adolescents. We observed a specific influence of the three factors in understanding antisocial behaviors, but also a cumulative effect explaining the types of offenses committed. Likewise, Waschbusch et al. (42) assessed the role of irritability and LPE/CU in externalizing and internalizing symptoms in children with attention-deficit/hyperactivity disorder, oppositional defiant disorder, and/or conduct disorder, but also in typically developing children. They observed a unique association between irritability and LPE/CU, and internalizing/externalizing symptoms, but also a cumulative effect on proactive aggression and on functional impairments (i.e., relationships with peers or adults, behavior in class, or self-esteem). Nevertheless, to the best of our knowledge, no studies considered both emotional features to predict long-term externalizing and/or adjustment problems within a transdiagnostic approach (i.e., focusing on a dimensional perspective of symptoms) among individuals with a residential care history into adulthood.

### The current study

As described above, anger/irritability and LPE/CU are two important emotional features associated with externalizing symptoms [e.g., (13, 14, 39, 40)]. Moreover, previous cross-sectional studies [e.g., (25, 42)] reported a negative cumulative effect (more negative impact in the presence of both risk factors) in different aspects of children’s behaviors and socialization. However, previous studies used a cross-sectional design. Thus, no longitudinal evidence is available. Moreover, these features have scarcely been studied in a vulnerable sample of young adults with a residential care history (at risk sample). Therefore, the aims of the present study are twofold: (a) to investigate the development of anger/irritability problems and LPE/CU traits from adolescence into adulthood among individuals with a history of residential care, and (b) to examine to what extend these emotional features predict externalizing problems and adjustment problems over this period. We hypothesize a reduction of symptoms over time. Furthermore, we expect a simple effect of the emotional features (direct effect of anger/irritability as well as LPE/CU) and a negative cumulative effect. Our findings will give more insight into the conjoint effect of both emotional factors from adolescence into adulthood on externalizing problems and adjustment problems in this high-risk population.

## Materials and methods

### Sample and procedure

The data were collected within the “Youth welfare trajectories: learning form experience” [“Jugendhilfverlaüfe: Aus Erfahung lernen” (JAEL)] project, a 10-year follow-up study of the “Clarification and Goal-Attainment in Child Welfare and Juvenile-Justice Institutions” [“Modellversuch zur Abklärung und Zielerreichung in stationären Massnahmen” (MAZ); (43) study]. The mean interval between baseline and follow-up was 9.7 years (range = 7–12 years). The study is a broad investigation of emotional and behavioral characteristics of youths who lived in institutions in Switzerland. Five hundred and ninety-two children, adolescents, and young adults from 64 Swiss child welfare and juvenile justice institutions were included at baseline. From the 592 participants at baseline, 511 youths agreed to be contacted again for the follow-up. However, 137 could not be reached (no trace found = 8; dead = 8; never answered to our solicitations = 121). Of the 374 participants reached, 231 consented to participate to the follow-up study, and 203 participants filled out all online questionnaires (accessed through a personal link *via* email). The socio-demographic characteristic of the sample is described in Table 1. Each participant gave their written consent after having received the information about the study. The procedure was approved by the Northwest- and Central-Swiss Ethics committee (*Ethikkommission Nordwest- und Zentralschweiz*). Then, each participant filled in self-report measures (see below for details).

**TABLE 1:** Socio-demographic characteristics (*n* = 203).

Variable		*N*	%
Gender	Women	80	33.9
Age at follow-up	Mean (SD)	26.30 (3.37)	
Birth place	Switzerland	178	80.9
	EU/UK	11	5.0
	Other	31	14.1
Education	Higher education	31	14.5
	Apprenticeship	114	53.3
	Mandatory school	59	27.6
	Unfinished mandatory school	10	4.7
Civil status	Single	200	90.9
	Married/partnership	15	6.8
	Divorced/separated	5	2.3
Having children	Yes	48	20.3
Living condition	By family/friends	132	61.4
	Alone	72	33.5
	Institution/jail	9	4.2
	Other	2	0.9
Income^a^	Employment/schooling/ apprenticeship	113	45.2
	Unemployment insurance	14	5.6
	Social insurance	59	23.6
	Disability insurance	34	13.6
	Help from relatives	28	11.2
	Other	2	0.8
Income satisfaction	Satisfied	61	28.6
	Just enough to live	59	27.7
	Not enough to live	93	43.7

^a^More than one response is possible.

### Measures

#### Outcome

Psychopathological symptoms were assessed with Youth Self-Report (YSR) (44) and the Young Adult Self-Report (YASR) (45). Each participant rated items describing common emotional and behavioral difficulties. Each response is rated on a three-point Likert scale from “*not true*” to “*often true.*” We used the externalizing symptoms score. Raw scores were transformed into T-scores to merge data from the different versions administered in function of the age of the participants. Moreover, to assess adjustment problems, the T-scores of externalizing symptoms and internalizing symptoms were averaged, as proposed in previous studies (13, 14). Higher scores indicate more symptoms. All Cronbach’s αs are higher than 0.84. This measure has been administered at baseline and at follow-up.

#### Predicting factors

The Affective scale of the Youth Psychopathic Traits Inventory (YPI) (46), a 50-item self-report questionnaire, was used to assess LPE/CU (baseline’s α = 0.84; follow-up’s α = 0.78). Each item is rated on a four-point Likert scale ranging from 0 = “*does not apply at all*” to 3 = “*apply very well.*” Higher scores indicate higher limited prosocial emotion/callous-unemotional traits. This measure has been administered at baseline and at follow-up.

The anger/irritability scale of the Massachusetts Youth Screening Instrument-Second Version (MAYSI-2) (47), a screening questionnaire with 52 questions, was used to assess anger/irritability (baseline’s α = 0.78; follow-up’s α = 0.83). Each item is rated either as 1 = “*yes*” or 0 = “*no*” considering the past month. Higher scores indicate more anger/irritability. This measure has been administered at baseline and at follow-up.

#### Early adversity

Early adversities (i.e., physical abuse, emotional abuse, physical neglect, emotional neglect, and sexual abuse) were collected at follow-up using the short form of the Child Trauma Questionnaire (CTQ-SF) (48), a 28-item self-report questionnaire. Each item is rated on a five-point Likert-type scale ranging from 0 = “*never true*” to 4 = “*very often true.*” We use the total score of the CTQ (Cronbach’s α = 0.90), with a higher score reflecting more adversity during childhood. This measure has been administered only at follow-up to encompass all adverse events.

### Data analysis

First, we reported descriptive statistics and compared the groups of youths included in the follow-up from those who dropped out from the study (i.e., sample attrition analysis). Also, we provide descriptive sample characteristics for sociodemographic variables. As we did not observe any systematic variation from sample attrition analyses (see below), we ran the analyses with the data at disposal without implementing missing data (i.e., case analyses). Parametric tests were conducted as the data suit Gaussian distribution (i.e., skewness and kurtosis and Q-Q plots were inspected). Repeated analyses of covariance (RM-ANCOVA) controlling for gender, age at baseline, and early adversities were computed to assess the differences between baseline and follow-up for the emotional features and psychopathological symptoms. Correlational analyses are provided in Supplementary File 1. Afterward, we conducted four multivariate linear regression models explaining externalizing symptoms or adjustment problems as well as their changes over the follow-up period by the predicting factors [i.e., anger/irritability, LPE/CU, changes (or delta), and their cumulative effect] as well as the control variables (i.e., gender, age at baseline, and early adversities). Changes over the follow-up period (or delta) scores were computed by subtracting the baseline scores from the follow-up scores, higher scores thus reflecting a worsening or an aggravation of symptoms with time.

## Results

### Descriptive and drop-out analyses

The socio-demographic characteristic of the sample is described in Table 1. The participants were on average of 26 years of age, and around 34% were women. Most of the youths were born in Switzerland (80.9%) and single at baseline (90.9%). Regarding their education, a minority dropped out of mandatory school and did not achieve basic education (4.7%). Concerning their incomes, almost half of the sample are employed or at school (45.2%) but an important part gets public financial support (42.8%). Almost half of the participants are not satisfied with their income (43.7%).

Table 2 shows descriptive data and drop-out (or attrition) analyses. No difference is observed between the youths included in the follow-up compared to those who dropped out from the study, indicating no apparent selection bias.

**TABLE 2:** Descriptive and drop-out analyses.

Variable		Drop out (*n* = 389)		Included (*n* = 203)		Drop out analyses
				
		*N*	%	*N*	%	*p* ^a^
Gender	Women	114	31.6	75	32.6	0.794
Nationality	Swiss	298	82.5	202	87.8	0.083
Type of institutional placement	Civil law	198	56.4	113	50.2	0.272
	Criminal law	89	25.4	59	26.2	
	Other	64	18.2	53	23.6	

		**Mean**	***SD***	**Mean**	***SD***	

Age at baseline		15.5	3.2	15.7	3.0	0.513
Early adversity		–	–	51.5	16.5	–
Anger/irritability at baseline		4.4	2.6	4.7	2.7	0.140
Limited prosocial emotion/callous-unemotional traits at baseline		32.4	7.1	33.0	8.2	0.207
Externalizing behaviors at baseline		60.8	10.0	61.2	11.2	0.476
Adjustment problems at baseline		59.3	8.9	59.3	10.1	0.876

^a^*P*-value from chi-square tests or multivariate analyses of variance, as appropriate.

### Changes over time

The RM-ANCOVA revealed no change over time (see Table 3).

**TABLE 3:** Baseline vs. follow-up comparisons.

Variable	Baseline		Follow-up		*p* ^a^
			
	*M*	*SD*	*M*	*SD*	
Anger/irritability	4.7	2.7	3.5	2.8	0.837
Limited prosocial emotion/callous-unemotional traits	33.0	8.2	29.4	6.3	0.175
Externalizing behaviors	61.2	11.2	53.1	9.8	0.214
Adjustment problems	59.3	10.1	53.1	9.5	0.595

^a^Results of repeated-measures analysis of covariance (controlling for age, gender, and early adversity).

### Externalizing behaviors

The regression analysis revealed that a significant proportion of the variance of externalizing behaviors at follow-up younger age at baseline, more externalizing symptoms at baseline, higher anger/irritability at baseline, and more worsening in anger/irritability with time are predictive of higher externalizing scores at follow-up, *F*(10, 119) = 8.24, *p* < 0.001, *R*^2^ = 0.431, and adjusted *R*^2^ = 0.378 (Table 4).

**TABLE 4:** Prediction of externalizing behaviors at follow-up.

	B	SE	β	*t*	*p* ^a^	CI (95%)	
Gender (0 = women, 1 = men)	–0.17	1.81	–0.01	–0.09	0.926	–3.76	3.42
**Age at baseline**	**–0.75**	**0.27**	**–0.21**	**–2.78**	**0.006**	**–1.28**	**–0.21**
Early adversity	0.05	0.05	0.08	1.01	0.314	–0.05	0.15
**Externalizing behavior at baseline**	**0.18**	**0.07**	**0.21**	**2.36**	**0.020**	**0.03**	**0.33**
**Anger/irritability**	**1.57**	**0.37**	**0.42**	**4.20**	**<0.001**	**0.83**	**2.30**
Limited prosocial emotion/callous-unemotional traits	0.14	0.13	0.12	1.05	0.294	–0.12	0.40
**Delta anger/irritability**	**1.72**	**0.27**	**0.57**	**6.28**	**<0.001**	**1.18**	**2.26**
Delta limited prosocial emotion/callous-unemotional traits	0.06	0.13	0.05	0.47	0.640	–0.20	0.33
Anger/irritability × Limited prosocial emotion/callous-unemotional traits	0.02	0.03	0.04	0.52	0.606	–0.05	0.08
Delta Anger/irritability × Limited prosocial emotion/callous-unemotional traits	–0.01	0.03	–0.03	–0.34	0.734	–0.07	0.05

Significant predictors are in bold.

^a^*P*-value resulting from multivariate linear regression analyses.

The regression analysis with the worsening with time in externalizing scores as outcome revealed that younger age at baseline, lower externalizing symptoms at baseline, higher anger/irritability at baseline, and a worsening of anger/irritability with time are associated with more worsening in externalizing scores over the 10-year follow-up, *F*(10, 119) = 19.19, *p* < 0.001, *R*^2^ = 0.638, and adjusted *R*^2^ = 0.604 (Table 5).

**TABLE 5:** Prediction of aggravation in externalizing behaviors over time (delta).

	B	SE	β	*t*	*p* ^a^	CI (95%)	
Gender (0 = women, 1 = men)	–0.17	1.81	–0.01	–0.09	0.926	–3.76	3.42
**Age at baseline**	**–0.75**	**0.27**	**–0.16**	**–2.78**	**0.006**	**–1.28**	**–0.21**
Early adversity	0.05	0.05	0.07	1.01	0.314	–0.05	0.15
**Externalizing behavior at baseline**	**–0.82**	**0.07**	**–0.78**	**–10.98**	**<0.001**	**–0.97**	**–0.67**
**Anger/irritability**	**1.57**	**0.37**	**0.34**	**4.20**	**<0.001**	**0.83**	**2.30**
Limited prosocial emotion/callous-unemotional traits	0.14	0.13	0.10	1.05	0.294	–0.12	0.40
**Delta anger/irritability**	**1.72**	**0.27**	**0.45**	**6.28**	**<0.001**	**1.18**	**2.26**
Delta limited prosocial emotion/callous-unemotional traits	0.06	0.13	0.04	0.47	0.640	–0.20	0.33
Anger/irritability × Limited prosocial emotion/callous-unemotional traits	0.02	0.03	0.03	0.52	0.606	–0.05	0.08
Delta Anger/irritability × Limited prosocial emotion/callous-unemotional traits	–0.01	0.03	–0.02	–0.34	0.734	–0.07	0.05

Significant predictors are in bold.

^a^*P*-value resulting from multivariate linear regression analyses.

### Adjustment problems

The regression analysis performed with adjustment problems at follow-up as outcome revealed that younger age at baseline, more anger/irritability, and a worsening in anger/irritability over time are related to more adjustment problems at follow-up, *F*(9, 119) = 9.72, *p* < 0.001, *R*^2^ = 0.471, and adjusted *R*^2^ = 0.423 (Table 6).

**TABLE 6:** Prediction of adjustment problems at follow-up.

	B	SE	β	*t*	*p* ^a^	CI (95%)	
Gender (0 = women, 1 = men)	–1.26	1.72	–0.06	–0.73	0.467	–4.67	2.15
**Age at baseline**	**–1.04**	**0.26**	**–0.29**	**–4.03**	**<0.001**	**–1.55**	**–0.53**
Early adversity	0.08	0.05	0.13	1.63	0.105	–0.02	0.17
Adjustment problems at baseline	0.10	0.08	0.11	1.28	0.203	–0.05	0.25
**Anger/irritability**	**1.36**	**0.36**	**0.37**	**3.82**	**<0.001**	**0.65**	**2.07**
Limited prosocial emotion/callous-unemotional traits	0.02	0.13	0.02	0.14	0.888	–0.23	0.27
**Delta anger/irritability**	**1.74**	**0.26**	**0.58**	**6.70**	**<0.001**	**1.23**	**2.26**
Delta limited prosocial emotion/callous-unemotional traits	–0.09	0.13	–0.08	–0.74	0.461	–0.35	0.16
Anger/irritability × Limited prosocial emotion/callous-unemotional traits	–0.02	0.03	–0.04	–0.53	0.594	–0.08	0.05
Delta Anger/irritability × Limited prosocial emotion/callous-unemotional traits	–0.01	0.03	–0.02	–0.32	0.751	–0.07	0.05

Significant predictors are in bold.

^a^*P*-value resulting from multivariate linear regression analyses.

The regression analysis with the aggravation of adjustment problems over time as outcome revealed that younger age at baseline, lower adjustment problems at baseline, more anger/irritability, and a worsening of anger/irritability over time are related to more aggravation in adjustment problems over the 10 years, *F*(10, 119) = 20.81, *p* < 0.001, *R*^2^ = 0.656, and adjusted *R*^2^ = 0.625 (Table 7).

**TABLE 7:** Prediction of impairment of adjustment problems (delta).

	B	SE	β	*t*	*p* ^a^	CI (95%)	
Gender (0 = women, 1 = men)	–1.26	1.72	–0.05	–0.73	0.467	–4.67	2.15
**Age at baseline**	**–1.04**	**0.26**	**–0.23**	**–4.03**	**<0.001**	**–1.55**	**–0.53**
Early adversity	0.08	0.05	0.11	1.63	0.105	–0.02	0.17
**Adjustment problems at baseline**	**–0.90**	**0.08**	**–0.80**	**–11.63**	**<0.001**	**–1.05**	**–0.75**
**Anger/irritability**	**1.36**	**0.36**	**0.30**	**3.82**	**<0.001**	**0.65**	**2.07**
Limited prosocial emotion/callous-unemotional traits	0.02	0.13	0.01	0.14	0.888	–0.23	0.27
**Delta anger/irritability**	**1.74**	**0.26**	**0.47**	**6.70**	**<0.001**	**1.23**	**2.26**
Delta limited prosocial emotion/callous-unemotional traits	–0.09	0.13	–0.06	–0.74	0.461	–0.35	0.16
Anger/irritability × Limited prosocial emotion/callous-unemotional traits	–0.02	0.03	–0.03	–0.53	0.594	–0.08	0.05
Delta Anger/irritability × Limited prosocial emotion/callous-unemotional traits	–0.01	0.03	–0.02	–0.32	0.751	–0.07	0.05

Significant predictors are in bold.

^a^*P*-value resulting from multivariate linear regression analyses.

## Discussion

The objectives of the present study were (a) to investigate the development of anger/irritability problems and LPE/CU from adolescence into adulthood among individuals with a history of residential care and (b) to what extend these emotions predict externalizing problems and its development and adjustment problems over this period.

First, the results of the study showed no changes in anger/irritability, LPE/CU, externalizing behaviors, and adjustment problems from adolescence into young adulthood, when most of them transited to other living styles (e.g., back to their family, with friend, with a partner or alone). Second, displaying more externalizing behaviors or adjustment problems, being younger, and showing more anger/irritability when in institution, as well as an increment in anger/irritability over time predicted more externalizing behaviors/adjustment problems 10 years later. Furthermore, an aggravation in externalizing symptoms/adjustment problems over the 10 year was found to be predicted by a younger age at baseline, a lower level of externalizing symptoms while in institution, but a higher level of anger/irritability and an aggravation of anger/irritability over time. LPE/CU was not found to be predictive of either externalizing symptoms or adjustment problems. Below these results are discussed in light of previous literature.

### Developmental aspects

Contrary to some studies in the general population (17, 22), we did not observe that psychopathological symptoms (i.e., externalizing behaviors or adjustment problems) are more prevalent in adolescence and decline in adulthood. Our study examined youths at risk, who live in residential care facilities which might account for the differences. Indeed, chronic stress has been observed to be associated with the development of maladaptive coping and regulation strategies which may facilitate the development of persistent externalizing behaviors as well as adjustment problems (5, 8–10).

Younger age at baseline was a significant predictor for higher externalizing and adjustment problems 10 years later, as well as regarding the aggravation of externalizing and adjustment problems during the study span. These results may be explained by the fact that younger participants were in late childhood/early adolescence at the baseline assessment, and due to their young age not engaged in delinquent activities yet (i.e., showed lower externalizing problems). With time, older participants naturally desisted from these activities, while younger participants were probably still in the peak of adolescence-driven behaviors at the follow-up assessment, which is why they reported more externalizing and adjustment problems than older participants at the follow-up.

### Predicting externalizing and adjustment problems and their aggravation over 10 years

In line with previous literature, anger regulation difficulties were related to externalizing symptoms and adjustment problems [e.g., (20, 25, 49–52)]. However, it is interesting to note that this factor is predictive for levels in externalizing and adjustment problems even 10 years later. Our study showed that a deterioration in anger/irritability with time was related to higher externalizing and adjustment problems and that it was also predictive of a deterioration of externalizing and adjustment problems 10 years later. These results are in line with evidence showing that irritability is related to long-term adverse outcomes, such as social impairment, low educational achievement, more delinquency, suicidality, and a higher prevalence of adult depression or anxiety (53–63). As the prevalence of mild irritability ranges from 20 to 30% (61, 64) and 1 to 3% for severe chronic forms of irritability (65–68), it should become a target of early assessment and early intervention.

### Clinical and preventive implications

Our results plead for systematic early intervention aiming at improving emotion regulation in institutions for adolescents showing anger/irritability problems. Adolescence is a pivotal period in terms of brain plasticity which offers a unique window of opportunity to alleviate future difficulties (69). In that sense, a consortium of experts (70) highlighted the existence of an urgent and important need to develop psychological interventions to manage emotion dysregulation, among other symptoms, to alleviate psychological sufferance. Furthermore, there is a need for methodologically sound studies evaluating innovative treatments based on theory and evidence and integrating an interdisciplinary perspective in the context of early intervention and prevention in at-risk populations should be a top priority (70). More specifically, matching proposed interventions to the specific deficits observed is essential to increase the benefits (71, 72). In addition, there is an urgent need to evaluate innovative interventions based on recent neurobiological findings in youth with externalizing symptoms (73), and from a criminological perspective, it is essential to develop a more integrative approach (74).

As a prevention to reduce anger dysregulation, residential care institutions may therefore implement individual or group programs aiming at an early identification of those problems. In particular, many different psychotherapeutical approaches exist to help individual regulate their anger and irritability. A scoping review (75) listed the existing interventions for self-regulatory control processes (including anger regulation and irritability) in youth with externalizing behaviors. However, many interventions involve parents, such as parent management training or cognitive-behavioral therapy [for a review see Sukhodolsky et al. (76)], which may be difficult in adolescents in institutions. A recent review of meta-analyses investigating usefulness of anger-related interventions reported that cognitive-behavioral therapies were the most effective for anger-related problems among adolescents and adults (77). In particular, currently the START NOW, an emotional regulation training, is under assessment in institutions in the residential care in Switzerland.^1^

From a neurobiological perspective, an interesting tool that might help to enhance anger management of the youths is heart rate variability biofeedback (HRVB). Indeed, HRVB allows, through simple breathing exercises coupled with positive emotions such as compassion, caring, or appreciation, to balance somatic (balance between the autonomic and central nervous system) and psychological processes (e.g., thoughts and emotions). This enhances the abilities of the individuals to better cope with the difficulties and challenges of their daily lives (78, 79) and improves their resilience (80). Numerous meta-analyses have demonstrated the effectiveness of this type of tool in reducing perceived stress, depression, and anxiety in adults (81–83) as well as in children and adolescents (84, 85). Likewise, neurofeedback seems a promising therapeutical approach to enhance emotion regulation skills. Indeed, neurofeedback refers to an brain training changing the number of synapses between neurons through learning and conditioning (86) improving the voluntary regulation of specific brain regions (87). It was demonstrated to be effective in improving self-control (88–91). Thus, such tools empower young people and may help them to improve their ability to cope with anger.

Recently, we have seen the emergence of new intervention methods applied mainly to the field of youth. These tools are presented in the form of mobile applications and allow the user to record a reaction (behavioral and/or cognitive) that he or she has had, at a specific time, in his or her environment and in response to a stimulus. These kinds of ecological momentary interventions (EMI) which refer to “*treatments that are provided to people during their everyday lives (i.e., in real time) and in natural settings (i.e., real world)*” (92) represent novel and preventive approaches, particularly adapted to young people. When such interventions are coupled and thus adapted by ecological momentary assessment (EMA), it allows the development of a just-in-time adaptive intervention (JITAI) providing a personalized intervention based on real-time assessment (93). JITAI provides the right type and amount of support at the right time (94). Technological advances offer the opportunity to create EMIs and, in particular, JITAI, which has the potential to modify the therapeutic treatment (95) by providing psychological intervention at the best time, in a real-world setting. A scoping review (96) was recently conducted on EMIs and retrieved 64 studies on EMIs. These interventions allow to capture intra-individual variability over time, resulting in a more realistic picture of the individuals’ emotional life (i.e., anger regulation) and allowing to provide the best advice or support at the best time (i.e., anger management).

Finally, another promising recent tool that might help these youths is virtual reality (VR). VR consists in immersing the patient in virtual environment to mimic real-life interactions and environment in order that the patient has the feeling of “real” experience (97). In particular, some of the advantages of VR treatment are high ecological validity, exposition to situation that are not possible in real-life situations, high level of control of the environment and the triggers of the problematic behaviors or symptoms (without endangering others), and high immersion in the situation (98). A systematic review demonstrated the value of this approach in the field of mental health treatment (99). Indeed, this systematic review demonstrated that VR treatment is as effective as classical cognitive-behavioral therapies and surpasses waiting list or control group. In particular, VR treatment allows the patient to learn practical skills needed in life outside the institutions (e.g., anger management) in a protected environment without leaving the institutions (100). More specifically, a promising treatment developed in this domain is the Virtual Reality Aggression Prevention Training (VRAPT). VRAPT has been shown to reduce hostility and non-planning impulsiveness as well as improve anger control at post-treatment, which was, however, not maintained at follow-up (101). However, more studies are warranted in this line to assess the potential of VR in helping youth for anger management.

### Limitations

The findings of our study should be interpreted in light of some limitations. First, the use of self-reported data only, relying on the individual introspective and self-observational capacities, is an important limitation to the study. Considering other sources of information (e.g., parents or caregivers) might have given us a more objective view of the phenomenon.

Second, the regression analysis explained a high proportion of the variance in externalizing and adjustment symptoms at 10-year follow-up and even more in the worsening of externalizing symptoms. However, an important proportion of the variance remains to be understood. Therefore, future studies should include other self-regulatory control processes (e.g., cognitive control, heart rate variability) to disentangle the effects of different predictors for externalizing and adjustment problems.

Finally, this study considered a high-risk sample of individuals formerly in residential care institutions, representing a heterogeneous sample. Therefore, our results cannot be generalized to other settings of care or clinical populations. Thus, more studies in different care settings, countries, and with different samples are needed.

## Conclusion

The findings of our study showed that anger/irritability and LPE/CU were high among children and adolescents in out-of-home care. Psychopathological symptoms diminished from adolescence in the transition to adulthood. Anger/irritability represents a risk factor for adjustment and externalizing problems in young adulthood among this high-risk population. The care systems and child and adolescent psychiatry are thus faced with the challenging objective to provide a successful transition from out-of-home care into an independent adult life for individuals with emotion regulation difficulties and externalizing problems. Together, our findings plead (a) for systematic assessments of anger/irritability problems and (b) early interventions which aim to improve anger/irritability in out-of-home placed adolescents. In such, anger/irritability might be seen as a temperamental characteristic that develops during childhood related to experiences and that might become a psychopathological symptom when it impairs functioning. However, more studies are needed to examine the underlying process sustaining the emergence of pathological irritability as well as to understand the effects of other factors influencing externalizing problems and adjustment problems with larger sample and across different care settings.

## Data availability statement

The raw data supporting the conclusions of this article will be made available by the authors, without undue reservation.

## Ethics statement

The studies involving human participants were reviewed and approved by Institutional Review Board of the State of Basel. Written informed consent to participate in this study was provided by the participants’ legal guardian/next of kin.

## Author contributions

JF, KS, CB, and MS were responsible for the project design, funding, and supervision of the whole study. SH, JP, DB, and SS participated in the data collection. SU conducted the data analyses. SU, SH, and JP interpreted data and drafted the different versions of the manuscript. All authors contributed critically to the numerous versions of the manuscript, contributed to the article, approved the submitted version, and agreed to be accountable for the content of the work.

## Footnotes

1
https://www.istartnow.ch

## References

[1] Jozefiak T Kayed NS Rimehaug T Wormdal AK Brubakk AM Wichstrøm L. Prevalence and comorbidity of mental disorders among adolescents living in residential youth care. *Eur Child Adolesc Psychiatry.* (2016) 25:33–47. 10.1007/s00787-015-0700-x 25749933 PMC4698296

[2] Bronsard G Alessandrini M Fond G Loundou A Auquier P Tordjman S The prevalence of mental disorders among children and adolescents in the child welfare system: a systematic review and meta-analysis. *Medicine.* (2016) 95:e2622. 10.1097/MD.0000000000002622 26886603 PMC4998603

[3] Fischer S Dölitzsch C Schmeck K Jörg MF Schmid M. Interpersonal trauma and associated psychopathology in girls and boys living in residential care. *Child Youth Serv Rev.* (2016) 67:203–11. 10.1016/j.childyouth.2016.06.013

[4] Schmid M Kölch M Fegert JM Schmeck K. *”Abschlussbericht für den Fachausschuss für die Modellversuche und das Bundesamt für Justiz Zusammenfassung der Wichtigsten Ergebnisse und Erkenntnisse des Modellversuchs Abklärung und Zielerreichung in Stationären Maßnahmen (MAZ).* Basel: Universitäre Psychiatrische Kliniken (2011).

[5] Collin-Vézina D Coleman K Milne L Sell J Daigneault I. Trauma experiences, maltreatment-related impairments, and resilience among child welfare youth in residential care. *Int J Ment Health Addict.* (2011) 9:577–89. 10.1007/s11469-011-9323-8

[6] Habersaat S Cyril B Klaus S Philippe S Eric F Jörg MF Gender differences in the relationship between strain, negative emotions and delinquent behaviors in institutionalized juveniles. *Deviant Behav.* (2020) 41:1113–24. 10.1080/01639625.2019.1596549

[7] Miller GE Chen E. Harsh family climate in early life presages the emergence of a proinflammatory phenotype in adolescence. *Psychol Sci.* (2010) 21:848–56. 10.1177/0956797610370161 20431047 PMC3207635

[8] Green JG McLaughlin KA Berglund PA Gruber MJ Sampson NA Zaslavsky AM Childhood adversities and adult psychiatric disorders in the national comorbidity survey replication I: associations with first onset of DSM-IV disorders. *Arch Gen Psychiatry.* (2010) 67:113–23. 10.1001/archgenpsychiatry.2009.186 20124111 PMC2822662

[9] McLaughlin KA Green JG Gruber MJ Sampson NA Zaslavsky AM Kessler RC. Childhood adversities and adult psychiatric disorders in the national comorbidity survey replication II: associations with persistence of DSM-IV disorders. *Arch Gen Psychiatry.* (2010) 67:124–32. 10.1001/archgenpsychiatry.2009.187 20124112 PMC2847359

[10] Goemans A van Geel M Vedder P. ‘Over three decades of longitudinal research on the development of foster children: a meta-analysis’. *Child Abuse Negl.* (2015) 42:121–34. 10.1016/j.chiabu.2015.02.003 25724659

[11] McElroy E Shevlin M Murphy J McBride O. ‘Co-occurring internalizing and externalizing psychopathology in childhood and adolescence: a network approach’. *Eur Child Adolesc Psychiatry.* (2018) 27:1449–57. 10.1007/s00787-018-1128-x 29520540

[12] Rutter M Kim-Cohen J Maughan B. ‘Continuities and discontinuities in psychopathology between childhood and adult life’. *J Child Psychol Psychiatry.* (2006) 47:276–95. 10.1111/j.1469-7610.2006.01614.x 16492260

[13] Donado C Friedrich Y Kossowsky J Locher C Koechlin H. ‘Exposure to parental depressive symptoms: a longitudinal analysis on the association with adolescents’ depressive symptoms and adjustment problems’. *J Dev Behav Pediatr.* (2020) 41:522–33. 10.1097/DBP.0000000000000820 32576787

[14] Koechlin H Donado C Berde CB Kossowsky J. ‘Effects of childhood life events on adjustment problems in adolescence: a longitudinal study’. *J Dev Behav Pediatr.* (2018) 39:629–41. 10.1097/DBP.0000000000000596 29944491

[15] Campbell SB. *Behavior Problems in Preschool Children: Clinical and Developmental Issues.* New-York, NY: Guilford Press (2006).

[16] Campbell SB. Behavior problems in preschool children: a review of recent research. *J Child Psychol Psychiatry.* (1995) 36:113–49. 10.1111/j.1469-7610.1995.tb01657.x 7714027

[17] Moffitt TE. ‘Life-course-persistent and adolescence-limited antisocial behavior: a 10-year research review and a research agenda. In: Moffitt TE editor. *Causes of Conduct Disorder and Juvenile Delinquency.* New York, NY: The Guildford Press (2003).

[18] Frick PJ Viding E. ‘Antisocial behavior from a developmental psychopathology perspective’. *Dev Psychopathol.* (2009) 21:1111–31. 10.1017/S0954579409990071 19825260

[19] Moffitt TE. ‘Life-course persistent versus adolescence-limited antisocial behavior. 2nd ed. In: Cicchetti D Cohen J editors. *Developemental Psychaphtology Risk, Disorder, and Adaptation.* New York, NY: Wiley (2006).

[20] Pardini DA Frick PJ. ‘Multiple developmental pathways to conduct disorder: current conceptualizations and clinical implications’. *J Can Acad Child Adolesc Psychiatry.* (2013) 22:20–5. 23390429 PMC3565711

[21] Frick PJ White SF. ‘Research review: the importance of callous-unemotional traits for developmental models of aggressive and antisocial behavior’. *J Child Psychol Psychiatry.* (2008) 49:359–75. 10.1111/j.1469-7610.2007.01862.x 18221345

[22] Fairchild G Passamonti L Hurford G Hagan CC von dem Hagen EA van Goozen SH Brain structure abnormalities in early-onset and adolescent-onset conduct disorder. *Am J Psychiatry.* (2011) 168:624–33. 10.1176/appi.ajp.2010.10081184 21454920

[23] Loeber R Pardini DA. ‘Neurobiology and the development of violence: common assumptions and controversies’. *Philos Trans Royal Soc Lond Ser B Biol Sci.* (2008) 363:2491–503. 10.1098/rstb.2008.0032 18434284 PMC2606713

[24] Herts KL McLaughlin KA Hatzenbuehler ML. ‘Emotion dysregulation as a mechanism linking stress exposure to adolescent aggressive behavior’. *J Abnorm Child Psychol.* (2012) 40:1111–22. 10.1007/s10802-012-9629-4 22466516 PMC3448707

[25] Urben S Stéphan P Habersaat S Francescotti E Fegert JM Schmeck K Examination of the importance of age of onset, callous-unemotional traits and anger dysregulation in youths with antisocial behaviors. *Eur Child Adolesc Psychiatry.* (2017) 26:87–97. 10.1007/s00787-016-0878-6 27277753

[26] McMahon RJ Witkiewitz K Kotler JS Group Conduct Problems Prevention Research. ‘Predictive validity of callous-unemotional traits measured in early adolescence with respect to multiple antisocial outcomes’. *J Abnorm Psychol.* (2010) 119:752–63. 10.1037/a0020796 20939651 PMC3760169

[27] Arsenio WF Cooperman S Lover A. ‘Affective predictors of preschoolers’ aggression and peer acceptance: direct and indirect effects’. *Dev Psychol.* (2000) 36:438–48. 10.1037/0012-1649.36.4.438 10902696

[28] Lengua LJ Kovacs EA. ‘Bidirectional associations between temparement and parenting and the prediction of adjustment problems in middle childhood’. *J Appl Dev Psychol.* (2005) 72:571–8.

[29] Rothbart MK Ahadi AS Hershey KL. ‘Temparement and social behavior in childhood’. *Merrill Palmer Q.* (1994) 40:21–39.

[30] Vogel AC Jackson JJ Barch DM Tillman R Luby JL. ‘Excitability and irritability in preschoolers predicts later psychopathology: the importance of positive and negative emotion dysregulation’. *Dev Psychopathol.* (2019) 31:1067–83. 10.1017/S0954579419000609 31109387 PMC7059859

[31] Whelan YM Leibenluft E Stringaris A Barker ED. Pathways from maternal depressive symptoms to adolescent depressive symptoms: the unique contribution of irritability symptoms. *J Child Psychol Psychiatry.* (2015) 56:1092–100. 10.1111/jcpp.12395 25665134 PMC4855627

[32] Hawes SW Perlman SB Byrd AL Raine A Loeber R Pardini DA. ‘Chronic anger as a precursor to adult antisocial personality features: the moderating influence of cognitive control’. *J Abnorm Psychol.* (2016) 125:64–74. 10.1037/abn0000129 26618654 PMC4701633

[33] Burke JD Loeber R Lahey BB. ‘Adolescent conduct disorder and interpersonal callousness as predictors of psychopathy in young adults’. *J Clin Child Adolesc Psychol.* (2007) 36:334–46. 10.1080/15374410701444223 17658978

[34] Frick PJ Ellis M. ‘Callous-unemotional traits and subtypes of conduct disorder’. *Clin Child Fam Psychol Rev.* (1999) 2:149–68. 11227072 10.1023/a:1021803005547

[35] Salekin RT Brannen DN Zalot AA Leistico AM Neumann CS. ‘Factor structure of psychopathy in youth – testing the applicability of the new four-factor model’. *Crim Justice Behav.* (2006) 33:135–57. 10.1177/0093854805284416

[36] Vasey MW Kotov R Frick PJ Loney BR. ‘The latent structure of psychopathy in youth: a taxometric investigation’. *J Abnorm Child Psychol.* (2005) 33:411–29. 10.1007/s10802-005-5723-1 16118989

[37] Byrd AL Loeber R Pardini DA. ‘Understanding desisting and persisting forms of delinquency: the unique contributions of disruptive behavior disorders and interpersonal callousness’. *J Child Psychol Psychiatry.* (2012) 53:371–80. 10.1111/j.1469-7610.2011.02504.x 22176342 PMC3708685

[38] Kahn RE Byrd AL Pardini DA. ‘Callous-unemotional traits robustly predict future criminal offending in young men’. *Law Hum Behav.* (2013) 37:87–97. 10.1037/b0000003 22731505 PMC3822438

[39] Blair RJR. Traits of empathy and anger: implications for psychopathy and other disorders associated with aggression. *Philos Trans Royal Soc B Biol Sci.* (2018) 373:20170155. 10.1098/rstb.2017.0155 29483341 PMC5832681

[40] Wakschlag LS Perlman SB Blair RJ Leibenluft E Briggs-Gowan MJ Pine DS. ‘The neurodevelopmental basis of early childhood disruptive behavior: irritable and callous phenotypes as exemplars’. *Am J Psychiatry.* (2018) 175:114–30. 10.1176/appi.ajp.2017.17010045 29145753 PMC6075952

[41] Hyde LW Burt SA Shaw DS Donnellan MB Forbes EE. Early starting, agressive, and/or callous-unemotional ? Examining the overlap and predictive utility of antisocial behavior subtypes. *J Abnorm Psychol.* (2015) 124:329–42. 10.1037/abn0000029 25603360 PMC4428963

[42] Waschbusch DA Baweja R Babinski DE Mayes SD Waxmonsky JG. ‘Irritability and limited prosocial emotions/callous-unemotional traits in elementary-school-age children’. *Behav Ther.* (2020) 51:223–37. 10.1016/j.beth.2019.06.007 32138934

[43] Schmid M Kölch M Joerg MF Schmeck K Maz-Team. *Abschlussbericht Modellversuch Abklärung und Zielerreichung in Stationären Massnahmen.* Bern: Bundesamt für Justiz (2013).

[44] Achenbach TM. *Manual for the Youth Self-Report and 1991 Profiles.* Burlington, VT: University of Vermont, Department of Psychiatry (1991).

[45] Achenbach TM. *Manual for the Young Adult Self-Report and Young Adult Behavior Checklist.* Burlington, VT: University of Vermont, Department of Psychiatry (1997).

[46] Andershed H Kerr M Stattin H Levander S. ‘Psychopathic traits in non referred youths: a new assessment tool. In: Blauuw E Sheridan L editors. *Psychopaths: Current International Perspectives.* The Hague: Elsevier (2002). 10.1037/t07576-000

[47] Grisso T Barnum R. *Massachusetts Youth Screening Intrument-Version 2 (MAYSI-2): User’s Manual and Technical Report.* Sarasota, FL: Professional Ressource Press (2014).

[48] Bernstein DP Ahluvalia T Pogge D Handelsman L. Validity of the childhood trauma questionnaire in an adolescent psychiatric population. *J Am Acad Child Adolesc Psychiatry.* (1997) 36:340–8. 10.1097/00004583-199703000-00012 9055514

[49] Beauchaine TP Gatzke-Kopp L Mead HK. Polyvagal theory and developmental psychopathology: emotion dysregulation and conduct problems from preschool to adolescence. *Biol Psychol.* (2007) 74:174–84. 10.1016/j.biopsycho.2005.08.008 17045726 PMC1801075

[50] Beauchaine TP Neuhaus E Brenner SL Gatzke-Kopp L. Ten good reasons to consider biological processes in prevention and intervention research. *Dev Psychopathol.* (2008) 20:745–74. 10.1017/S0954579408000369 18606030 PMC2690981

[51] Gross JJ Jazaieri H. ‘Emotion, emotion regulation, and psychopathology: an affective science perspective’. *Clin Psychol Sci.* (2014) 2:387–401. 10.1177/2167702614536164

[52] Farb NAS Anderson AK Irving JA Segal ZV. ‘Mindfulness interventions and emotion regulation. In: Gross JJ editor. *Handbook of Emotion Regulation.* New-York, NY: The Guilford Press (2014).

[53] Brotman MA Kircanski K Leibenluft E. ‘Irritability in children and adolescents’. *Annu Rev Clin Psychol.* (2017) 13:317–41. 10.1146/annurev-clinpsy-032816-044941 28482689 PMC13292837

[54] Brotman MA Kircanski K Stringaris A Pine DS Leibenluft E. ‘Irritability in youths: a translational model’. *Am J Psychiatry.* (2017) 174:520–32. 10.1176/appi.ajp.2016.16070839 28103715 PMC13335380

[55] Fichter MM Kohlboeck G Quadflieg N Wyschkon A Esser G. ‘From childhood to adult age: 18-year longitudinal results and prediction of the course of mental disorders in the community’. *Soc Psychiatry Psychiatr Epidemiol.* (2009) 44:792–803. 10.1007/s00127-009-0501-y 19212695

[56] Stringaris A Cohen P Pine DS Leibenluft E. ‘Adult outcomes of youth irritability: a 20-year prospective community-based study’. *Am J Psychiatry.* (2009) 166:1048–54. 10.1176/appi.ajp.2009.08121849 19570932 PMC2791884

[57] Brotman DJ. Presentation of diagnostic test accuracy. *Ann Intern Med.* (2006) 144:221;authorrely21–2. 10.7326/0003-4819-144-3-200602070-00020 16461974

[58] Deveney CM Hommer RE Reeves E Stringaris A Hinton KE Haring CT ‘A prospective study of severe irritability in youths: 2- and 4-year follow-up’. *Depress Anxiety.* (2015) 32:364–72. 10.1002/da.22336 25504765 PMC10530700

[59] Stringaris A Zavos H Leibenluft E Maughan B Eley TC. ‘Adolescent irritability: phenotypic associations and genetic links with depressed mood’. *Am J Psychiatry.* (2012) 169:47–54. 10.1176/appi.ajp.2011.10101549 22193524 PMC3660701

[60] Copeland WE Shanahan L Egger H Angold A Costello EJ. ‘Adult diagnostic and functional outcomes of DSM-5 disruptive mood dysregulation disorder’. *Am J Psychiatry.* (2014) 171:668–74. 10.1176/appi.ajp.2014.13091213 24781389 PMC4106474

[61] Pickles A Aglan A Collishaw S Messer J Rutter M Maughan B. ‘Predictors of suicidality across the life span: the Isle of Wight study’. *Psychol Med.* (2010) 40:1453–66. 10.1017/S0033291709991905 19939326

[62] Sorcher LK Goldstein BL Finsaas MC Carlson GA Klein DN Dougherty LR. ‘Preschool irritability predicts adolescent psychopathology and functional impairment: a 12-year prospective study’. *J Am Acad Child Adolesc Psychiatry.* (2022) 61:554–64.e1. 10.1016/j.jaac.2021.08.016 34481916 PMC9951107

[63] Vidal-Ribas P Brotman MA Valdivieso I Leibenluft E Stringaris A. The status of irritability in psychiatry: a conceptual and quantitative review’. *J Am Acad Child Adolesc Psychiatry.* (2016) 55:556–70. 10.1016/j.jaac.2016.04.014 27343883 PMC4927461

[64] Collishaw S Maughan B Natarajan L Pickles A. ‘Trends in adolescent emotional problems in England: a comparison of two national cohorts twenty years apart’. *J Child Psychol Psychiatry.* (2010) 51:885–94. 10.1111/j.1469-7610.2010.02252.x 20497281

[65] Brotman MA Schmajuk M Rich BA Dickstein DP Guyer AE Costello EJ ‘Prevalence, clinical correlates, and longitudinal course of severe mood dysregulation in children’. *Biol Psychiatry.* (2006) 60:991–7. 10.1016/j.biopsych.2006.08.042 17056393

[66] Copeland WE Angold A Costello EJ Egger H. ‘Prevalence, comorbidity, and correlates of DSM-5 proposed disruptive mood dysregulation disorder’. *Am J Psychiatry.* (2013) 170:173–9. 10.1176/appi.ajp.2012.12010132 23377638 PMC3573525

[67] Laporte PP Matijasevich A Munhoz TN I Santos S Barros AJD Pine DS ‘Disruptive mood dysregulation disorder: symptomatic and syndromic thresholds and diagnostic operationalization’. *J Am Acad Child Adolesc Psychiatry.* (2021) 60:286–95. 10.1016/j.jaac.2019.12.008 32004697 PMC9073144

[68] Munhoz TN I Santos S Barros AJD Anselmi L Barros FC Matijasevich A. ‘Perinatal and postnatal risk factors for disruptive mood dysregulation disorder at age 11: 2004 pelotas birth cohort study’. *J Affect Disord.* (2017) 215:263–8. 10.1016/j.jad.2017.03.040 28347949 PMC5408904

[69] Fuhrmann D Knoll LJ Blakemore SJ. ‘Adolescence as a sensitive period of brain development’. *Trends Cogn Sci.* (2015) 19:558–66. 10.1016/j.tics.2015.07.008 26419496

[70] Holmes EA Ghaderi A Harmer CJ Ramchandani PG Cuijpers P Morrison AP ‘The lance psychiatry commission on psychological treatments research in tomorrow’s science’. *Lancet.* (2018) 5:237–86. 10.1016/S2215-0366(17)30513-8 29482764

[71] Baskin-Sommers AR Curtin JJ Newman JP. ‘Altering the cognitive-affective dysfunctions of psychopathic and externalizing offender subtypes with cognitive remediation’. *Clin Psychol Sci.* (2015) 3:45–57. 10.1177/2167702614560744 25977843 PMC4426343

[72] Glenn AL McCauley KE. ‘How biosocial research can improve intervention for antiosocial behavior’. *J Contem Crim Justice.* (2019) 35:103–19. 10.1177/1043986218810608

[73] Bootsman F. Neurobiological intervention and prediction of treatment outcome in the juvenile criminal justice system. *J Crim Just.* (2019) 65:101554. 10.1016/j.jcrimjus.2018.05.001 30875009

[74] Wilson LC Scarpa A. ‘Criminal behavior: the need of an integrative approach that incorporates biological influences’. *J Contemp Crim Justice.* (2012) 28:366–81. 10.1177/1043986212450232

[75] Constanty L Lepage C Rosselet Amoussou J Wouters E Decoro V De-Paz L Non-pharmaceutical interventions for self-regulatory failures in adolescents suffering from externalizing symptoms: a scoping review. *Biomedicines.* (2021) 9:1081. 10.3390/biomedicines9091081 34572267 PMC8466021

[76] Sukhodolsky DG Smith SD McCauley SA Ibrahim K Piasecka JB. ‘Behavioral interventions for anger, irritability, and aggression in children and adolescents’. *J Child Adolesc Psychopharmacol.* (2016) 26:58–64. 10.1089/cap.2015.0120 26745682 PMC4808268

[77] Lee AH DiGiuseppe R. ‘Anger and aggression treatments: a review of meta-analyses’. *Curr Opin Psychol.* (2018) 19:65–74. 10.1016/j.copsyc.2017.04.004 29279226

[78] Lehrer P Gevirtz R. ‘Heart rate variability biofeedback: how and why does it work?’. *Front Psychol.* (2014) 5:756. 10.3389/fpsyg.2014.00756 25101026 PMC4104929

[79] McCraty R. ‘Coherence: bridging personal, social and global health’. *Activit Nerv Super Rediv.* (2011) 53:85–102.

[80] Lehrer P Kaur K. ‘Heart rate variability biofeedback promotes general resilience’. *Psychos Med.* (2020) 82:A175–6.

[81] Goessl VC Curtiss JE Hofmann SG. ‘The effect of heart rate variability biofeedback training on stress and anxiety: a meta-analysis’. *Psychol Med.* (2017) 47:2578–86. 10.1017/S0033291717001003 28478782

[82] Lehrer P Kaur K Sharma A Shah KB Huseby R Bhavsar J ‘Heart rate variability biofeedback improves emotional and physical health and performance: a systematic review and meta analysis’. *Appl Psychophysiol Biofeedback.* (2020) 45:109–29. 10.1007/s10484-020-09466-z 32385728

[83] Pizzoli SFM Marzorati C Gatti D Monzani D Mazzocco K Pravettoni G. A meta-analysis on heart rate variability biofeedback and depressive symptoms. *Sci Rep.* (2021) 11:6650. 10.1038/s41598-021-86149-7 33758260 PMC7988005

[84] Dormal V Vermeulen N Mejias S. ‘Is heart rate variability biofeedback useful in children and adolescents? A systematic review’. *J Child Psychol Psychiatry.* (2021) 62:1379–90. 10.1111/jcpp.13463 34155631

[85] Thabrew H Ruppeldt P Sollers JJ III. ‘Systematic review of biofeedback interventions for addressing anxiety and depression in children and adolescents with long-term physical conditions’. *Appl Psychophysiol Biofeedback.* (2018) 43:179–92. 10.1007/s10484-018-9399-z 29946920

[86] Kandel ER Schwartz JH Jessell TM. *Principles of Neural Science.* New York, NY: McGraw-hill, Health Professions Division (2000).

[87] Sulzer J Haller S Scharnowski F Weiskopf N Birbaumer N Blefari ML ‘Real-time fMRI neurofeedback: progress and challenges’. *Neuroimage.* (2013) 76:386–99. 10.1016/j.neuroimage.2013.03.033 23541800 PMC4878436

[88] Abdian H Rezaei M Eskandari Z Ramezani S Pirzeh R Dadashi M. ‘The effect of quantitative electroencephalography-based neurofeedback therapy on anxiety, depression, and emotion regulation in people with generalized anxiety disorder’. *Basic Clin Neurosci.* (2021) 12:281–90. 10.32598/bcn.12.2.2378.1 34925724 PMC8672673

[89] Faridnia M Shojaei M Rahimi A. ‘The effect of neurofeedback training on the anxiety of elite female swimmers’. *Ann Biol Res.* (2012) 3:1020–8.

[90] Sulzer J Sitaram R Blefari ML Kollias S Birbaumer N Stephan KE ‘Neurofeedback-mediated self-regulation of the dopaminergic midbrain’. *Neuroimage.* (2013) 83:817–25. 10.1016/j.neuroimage.2013.05.115 23791838

[91] Young KD Zotev V Phillips R Misaki M Drevets WC Bodurka J. ‘Amygdala real-time functional magnetic resonance imaging neurofeedback for major depressive disorder: a review’. *Psychiatry Clin Neurosci.* (2018) 72:466–81. 10.1111/pcn.12665 29687527 PMC6035103

[92] Heron KE Smyth JM. ‘Ecological momentary interventions: incorporating mobile technology into psychosocial and health behaviour treatments’. *Br J Health Psychol.* (2010) 15:1–39. 10.1348/135910709X466063 19646331 PMC2800172

[93] Patrick K Intille SS Zabinski MF. ‘An ecological framework for cancer communication: implications for research’. *J Med Internet Res.* (2005) 7:e23. 10.2196/jmir.7.3.e23 15998614 PMC1550654

[94] Nahum-Shani I Smith SN Spring BJ Collins LM Witkiewitz K Tewari A Just-in-time adaptive interventions (JITAIs) in mobile health: key components and design principles for ongoing health behavior support. *Ann Behav Med.* (2017) 52:446–62. 10.1007/s12160-016-9830-8 27663578 PMC5364076

[95] Shiffman S Stone AA Hufford MR. ‘Ecological momentary assessment’. *Annu Rev Clin Psychol.* (2008) 4:1–32. 10.1146/annurev.clinpsy.3.022806.091415 18509902

[96] Balaskas A Schueller SM Cox AL Doherty G. Ecological momentary interventions for mental health: a scoping review. *PLoS One.* (2021) 16:e0248152. 10.1371/journal.pone.0248152 33705457 PMC7951936

[97] Riva G Banos RM Botella C Mantovani F Gaggioli A. Transforming experience: the potential of augmented reality and virtual reality for enhancing personal and clinical change. *Front Psychiatry.* (2016) 7:164. 10.3389/fpsyt.2016.00164 27746747 PMC5043228

[98] Fromberger P Jordan K Muller JL. ‘Virtual reality applications for diagnosis, risk assessment and therapy of child abusers’. *Behav Sci Law.* (2018) 36:235–44. 10.1002/bsl.2332 29520819

[99] Valmaggia LR Latif L Kempton MJ Rus-Calafell M. ‘Virtual reality in the psychological treatment for mental health problems: an systematic review of recent evidence’. *Psychiatry Res.* (2016) 236:189–95. 10.1016/j.psychres.2016.01.015 26795129

[100] Kip H Kelders SM Weerink K Kuiper A Brüninghoff I Bouman YHA Identifying the added value of virtual reality for treatment in forensic mental health: a scenario-based, qualitative approach. *Front Psychol.* (2019) 10:406. 10.3389/fpsyg.2019.00406 30873093 PMC6400887

[101] Klein Tuente S Bogaerts S Bulten E Keulen-de Vos M Vos M Bokern H Virtual reality aggression prevention therapy (VRAPT) versus waiting list control for forensic psychiatric inpatients: a multicenter randomized controlled trial. *J Clin Med.* (2020) 9:2258. 10.3390/jcm9072258 32708637 PMC7409015

