# Erratum: Examination of the importance of anger/irritability and limited prosocial emotion/callous-unemotional traits to understand externalizing symptoms and adjustment problems in adolescence: A 10-year longitudinal study

**DOI:** 10.3389/fpsyt.2023.1175499

**Published:** 2023-03-10

**Authors:** Frontiers Production Office

**Affiliations:** Frontiers Media SA, Lausanne, Switzerland

**Keywords:** emotions, self-regulation (SC 23180), adjustment problems, adolescents, young adults, longitudinal design

An omission to the funding section of the original article was made in error. The following sentence has been added: “Open access funding was provided by the University of Lausanne.”

The publisher apologizes for this error. The original article has been updated.

