# Supplementary material for: Examination of the importance of anger/irritability and limited prosocial emotion/callous-unemotional traits to understand externalizing symptoms and adjustment problems in adolescence: A 10-year longitudinal study
Source: Front Psychiatry. 2022 Sep 29;13:939603. doi: 10.3389/fpsyt.2022.939603 (PMC9556640; doi:10.3389/fpsyt.2022.939603)
Supplement: Supplementary file 1 [file Table_1.DOCX]

Supplementary Material

**Correlational analyses**

*Data analyses*

Then, we conducted correlational analyses between the outcomes (i.e., level of externalizing symptoms and adjustment problems as well as their changes), the predictors (i.e., anger/irritability, LPE/CU, their moderating effect, and their changes) as well as the control variables (i.e., gender and trauma). To do so, Bravais-Pearson coefficient of correlations were computed as data suit Gaussian-like distribution.

*Associations*

The Bravais-Pearson coefficients of correlations are summarized in Table S1 below.

Female experienced less childhood adversities, reported lower externalizing and adjustment problems, lower anger/irritability, and higher LPE/CU scores both at baseline and at follow-up. . More childhood adversities is related to higher externalizing and adjustment problems score both at baseline and at follow-up. Moreover, a higher level of childhood adversities is related to higher anger/irritability and LPE/CU scores. Externalizing at baseline is correlated positively with adjustment problems at baseline and at follow-up, anger/irritability, LPE/CU scores at baseline and externalizing symptoms at follow-up, but negatively with their changes. Adjustment problems at baseline follow the same pattern. Anger/irritability is related positively to LPE/CU, externalizing and adjustment problems at follow-up, but negatively with the delta scores. LPE/CU is positively related to externalizing at follow-up and with changes in LPE/CU as well as externalizing symptoms. Externalizing at follow-up is positively related to adjustment problems, delta anger/irritability and delta EXT as well as delta of adjustment problems. Adjustment problems at follow-up is positively related to delta anger/irritability, as well as, the delta of externalizing and of adjustment problems. Delta anger/irritability is positively correlated to delta of externalizing and of adjustment problems. Delta LPE/CU is positively associated to delta externalizing.

**Table S1** Bravais-Pearson coefficients

|  | Gender^a^ | Age at baseline | Trauma | Externalizing at baseline | Adjustment problems at baseline | Anger/irritability | LPE/CU | Externalizing at follow up | Adjustment problems at follow up | Delta Anger/irritability | Delta LPE/CU | Delta Externalizing |
| --- | --- | --- | --- | --- | --- | --- | --- | --- | --- | --- | --- | --- |
| Early adversities | -.36^**^ | -.07 |  |  |  |  |  |  |  |  |  |  |
| Externalizing at baseline | -.22^**^ | -.07 | .27^**^ |  |  |  |  |  |  |  |  |  |
| Adjustment problems at baseline | -.29^**^ | -.30^**^ | .31^**^ | .89^**^ |  |  |  |  |  |  |  |  |
| Anger/irritability | -.19^**^ | -.02 | .32^**^ | .50^**^ | .50^**^ |  |  |  |  |  |  |  |
| LPE/CU | .31^**^ | .03 | .04 | .33^**^ | .17^*^ | .18^**^ |  |  |  |  |  |  |
| EXT at follow up | -.14^*^ | -.22^*^ | .31^**^ | .42^**^ | .36^**^ | .28^**^ | .17^*^ |  |  |  |  |  |
| Adjustment problems at follow up | -.21^**^ | -.31** | .38^**^ | .39^**^ | .41^**^ | .26^**^ | .08 | .89^**^ |  |  |  |  |
| Delta Anger/irritability | .09 | .02 | -.02 | -.18^*^ | -.17^*^ | -.58^**^ | -.13 | .23^**^ | .29^**^ |  |  |  |
| Delta LPE/CU | -.07 | -.11 | -.03 | -.31^**^ | -.20^**^ | -.21^**^ | -.69^**^ | -.07 | -.08 | .09 |  |  |
| Delta Externalizing | .14 | -.11 | -.01 | -.60^**^ | -.55^**^ | -.21^**^ | -.17^*^ | .47^**^ | .39^**^ | .38^**^ | .24^**^ |  |
| Delta adjustment problems | .14 | -.15 | .02 | -.48^**^ | -.57^**^ | -.22^**^ | -.08 | .45^**^ | .51^**^ | .42^**^ | .11 | .87^**^ |

* *p* <.05, ** *p* <.01. LPE/CU: Limited prosocial emotion / callous-unemotional traits. Delta: Change score

^a^ 0=women, 1= men.
